# Development of a Biosensor Based on Angiotensin-Converting Enzyme II for Severe Acute Respiratory Syndrome Coronavirus 2 Detection in Human Saliva

**DOI:** 10.3389/fsens.2022.917380

**Published:** 2022-07-13

**Authors:** Geisianny Moreira, Lisseth Casso-Hartmann, Shoumen Palit Austin Datta, Delphine Dean, Eric McLamore, Diana Vanegas

**Affiliations:** 1 Department of Environmental Engineering and Earth Sciences, Clemson University, Clemson, SC, United States; 2 Global Alliance for Rapid Diagnostics, Michigan State University, Cambridge, MI, United States; 3 Medical Device (MDPnP) Interoperability and Cybersecurity Labs, Biomedical Engineering Program, Department of Anesthesiology, Massachusetts General Hospital, Harvard Medical School, Cambridge, MA, United States; 4 MIT Auto-ID Labs, Department of Mechanical Engineering, Massachusetts Institute of Technology, Cambridge, MA, United States; 5 Center for Innovative Medical Devices and Sensors (REDDI Lab), Clemson University, Clemson, SC, United States; 6 Department of Bioengineering, Clemson University, Clemson, SC, United States; 7 Department of Agricultural Sciences, Clemson University, Clemson, SC, United States

**Keywords:** betacoronavirus, human ACE2, attenuated virus, biosensing, LIG electrodes, saliva diagnostics

## Abstract

Severe acute respiratory syndrome coronavirus 2 (SARS-CoV-2) is the novel coronavirus responsible for COVID-19. Infection in humans requires angiotensin-converting enzyme II (hACE2) as the point of entry for SARS-CoV-2. PCR testing is generally definitive but expensive, although it is highly sensitive and accurate. Biosensor-based monitoring could be a low-cost, accurate, and non-invasive approach to improve testing capacity. We develop a capacitive hACE2 biosensor for intact SARS-CoV-2 detection in saliva. Laser-induced graphene (LIG) electrodes were modified with platinum nanoparticles. The quality control of LIG electrodes was performed using cyclic voltammetry. Truncated hACE2 was used as a biorecognition element and attached to the electrode surface by streptavidin–biotin coupling. Biolayer interferometry was used for qualitative interaction screening of hACE2 with UV-attenuated virions. Electrochemical impedance spectroscopy (EIS) was used for signal transduction. Truncated hACE2 binds wild-type SARS-CoV-2 and its variants with greater avidity than human coronavirus (common cold virus). The limit of detection (LoD) is estimated to be 2,960 copies/ml. The detection process usually takes less than 30 min. The strength of these features makes the hACE2 biosensor a potentially low-cost approach for screening SARS-CoV-2 in non-clinical settings with high demand for rapid testing (for example, schools and airports).

## INTRODUCTION

The ongoing global pandemic of coronavirus disease (COVID-19) began around December 2019 and, to the point of writing, has caused nearly 490 million confirmed cases reported worldwide (World Health Organization, WHO). The severe acute respiratory syndrome coronavirus 2 (SARS-CoV-2) was identified as the microorganism responsible for the COVID-19 pandemic. Current data on SARS-CoV-2 suggest an evolution rate of 10^−3^ nucleotides^−1^ year^−1^ and a mutation rate of 10^−6^ nucleotides^−1^ cycle^−1^ (Bar-On et al., 2020; Singh and Yi, 2021). SARS-CoV-2 has a genome of approximately 30 kb, which encodes four structural proteins. These include spike protein (S), envelope protein (E), membrane protein (M), nucleocapsid protein (N), and several non-structural open reading frames (ORFs) (Singh and Yi, 2021). Due to the mutation capacity of the RNA virus, it is important to select robust biorecognition mechanisms to reduce the risk of false-negative detection. There are surface proteins on SARS-CoV-2 (e.g., S, E, and M proteins) that could potentially be targets for detection of the intact virion. Among these, the most prominent is the S protein trimer. During infection, human angiotensin-converting enzyme II (hACE2) is the first point of contact with S protein, mediating entry of SARS-CoV-2 (Shang et al., 2020). Downstream fusion of the viral and host membranes involves a cascade of events that require other proteins. Subunit 1 (S1) of the viral S protein is the specific portion that binds to hACE2 during the initial phase of cellular infection (Lan et al., 2020; Ni et al., 2020; Samavati and Uhal, 2020; Yan et al., 2020). SARS-CoV-2 attachment with the host cell involves simultaneous interactions of two S-glycoprotein trimers to the hACE2 dimer (Xu et al., 2021). Subsequently, S protein subunit 1 and subunit 2 are cleaved by the protease TMPRSS2, and a cascade of events then leads to membrane fusion for cell entry (Benton et al., 2020; Hoffmann et al., 2020a; Hoffmann et al., 2020b). Given the affinity of hACE2 for S protein, it is rational to explore hACE2 as a bioreceptor for development of bio detectors and biosensors (Lima et al., 2021; Vezza et al., 2021; Büyüksünetçi et al., 2022).

SARS-CoV-2 analytical detection has been demonstrated using a wide variety of techniques, including MALDI-TOF mass spectrometry (Hernandez et al., 2021; Preianò et al., 2021; Han et al., 2022; Sivanesan et al., 2022), radiolabeled immunosensors (Pirovano et al., 2021), and reverse transcriptase PCR (Ham et al., 2022), among others. Current gold standard laboratory testing of SARS-CoV-2 infections is based on reverse transcriptase–polymerase chain reaction (RT-PCR) (Wang W. et al., 2020). Laboratory methods, while extremely accurate and high throughput, have several shortcomings when applied in non-clinical and decentralized settings. The high instrument cost, need for specialized reagents and complex laboratory facilities, and lack of mobility are major challenges of existing technologies, thus limiting the testing capacity in certain areas (e.g., rural areas and low-income communities). Even in high-end clinical settings, existing sophisticated tools may limit testing capacity in resource-constrained communities with high demand (Giri and Rana, 2020; Tang et al., 2020). Potentially low-cost biosensors may be designed to fill this technology gap and facilitate rapid point-of-need screening (McLamore et al., 2019; Morgan et al., 2020; McLamore et al., 2021; McLamore et al., 2022).

To be widely adopted, biosensor-based monitoring should be low-cost, accurate, and non-invasive to the users. Within the types of analytical devices that have been explored for rapid diagnostics, electrochemical systems are amongst the most versatile. Advantages and disadvantages of electrochemical biosensors for virus detection (including immunosensors for SARS-CoV-2) are summarized by Campos-Ferreira et al. (2021), but this review did not address specific challenges associated with use of non-invasive saliva for diagnostics.

During the infectious window, the viral load of SARS-CoV-2 in saliva ranges from 10^4^ to 10^11^ RNA/ml (Bar-On et al., 2020). The Center for Disease Control and Prevention (CDC) recommends a limit of detection of 10^3^ copies/ml (Ct value <35) as threshold for diagnostic purposes. This recommendation is based on the use of real-time RT-PCR targeting the N1 gene (Arnaout et al., 2020; Centers for Disease Control and Prevention, 2021). Saliva is commonly used as a diagnostic medium in clinical and non-clinical settings (Malathi et al., 2014; Roi et al., 2019; Roi et al., 2020). However, there are many challenges and benefits of saliva monitoring in SARS-CoV-2 surveillance (Sri Santosh et al., 2020; Khemka et al., 2021). To date, most electrochemical biosensors for SARS-CoV-2 are limited to proof-of-concept demonstration in buffers. Demonstration of electrochemical detection in real saliva is challenging due to the complex and variable composition of the sample and the target, which may induce matrix effects and interference with electrochemical signal transduction.

There is a library of bio-recognition structures that could be explored for targeting S protein on SARS-CoV-2 in saliva. The classes of bioreceptors that bind to S protein include peptides (Wang J. et al., 2021; Yang et al., 2022), antibodies (Yousefi et al., 2021), aptamers (Song et al., 2020; Peinetti et al., 2021), and enzymes (Zost et al., 2020; Georgas et al., 2022). Among these, peptides, antibodies, and aptamers bind specific regions in the S protein but may lose clinical sensitivity due to mutations in S protein. hACE2 is a unique bio-recognition element because S protein mutants must still bind to hACE2 if the virus wishes to enter the human cell. A detection system using hACE2 (or truncated hACE2) as the bioreceptor may act as a “platform” for a family of SARS-CoV viruses. Through hACE2-based sensing could be prone to false-positive results when screening for a specific viral strain, these “generic” results are useful given the antigenic drift observed in the family of viruses that use hACE2-mediated infection (such as SARS-CoV-2).

As reviewed by numerous groups, there are many options for electrode platforms that may be used in development of hACE2 biosensors (Lucarelli et al., 2004; Rasouli et al., 2018; Białobrzeska et al., 2020; Zhao et al., 2021). Among these, laser-induced graphene (LIG) is an electrode material that is relatively facile, low-cost, and potentially more sustainable for manufacturing. LIG represents attractive advantages over other types of carbon electrodes with similar performance capabilities (Wang L. et al., 2020; Vivaldi et al., 2021; Dixit and Singh, 2022). We describe the development of a capacitive biosensor for intact SARS-CoV-2 detection in saliva. The electrode platform is based on LIG electrodes biofunctionalized with truncated hACE2.

## MATERIAL AND METHODS

### Materials and Reagents

Commercial electrical grade polyimide (Kapton film, 0.005” thickness) was obtained from McMaster-Carr (Elmhurst, IL, United States). Potassium ferrocyanide [K_4_Fe(CN)_6_], potassium ferricyanide [K₃Fe(CN)₆], and Tween 20™ were obtained from Fisher Scientific (Waltham, MA, United States). Potassium chloride (KCl), chloroplatinic acid solution (H_2_PtCl_6_), and lead (II) acetate trihydrate [Pb(CH_3_CO_2_)_2_·3H_2_O] were purchased from Sigma Aldrich (St. Louis, MO, United States). Sodium chloride/sodium bicarbonate physiological home-use solution (Sinus Rinse™, NeilMed) was obtained from a local pharmacy.

Truncated human ACE2 (C-terminal 6X his-tag; HEK293 cells expression system; 83 kDa; >95% purity; catalog number: 230–30165) was purchased from RayBiotech, Inc. (Norcross, GA, United States). EZ-Link™ NHS-PEG4-Biotin, No-Weigh™ Format, and Zeba™ Spin desalting column (7k MWCO) were obtained from Fisher Scientific (Waltham, MA, United States). Recombinant spike protein RBD domain (C-terminal 6X his-tag; Sf9 insect cells expression system; 25 kDa; >90% purity; catalog number: Z03479) was obtained from Genscript, Inc. (Piscataway, NJ, United States). Recombinant streptavidin (N-terminal 6X his-tag; *Escherichia coli* expression system; 18 kDa; >95% purity, catalog number: ab78833) was purchased from Abcam, Inc. (Cambridge, United Kingdom). Attenuated SARS-CoV-2 virus samples were obtained from the University of California San Diego through the NIH RADx-rad Diagnostics Core Center (DCC) (Supplementary Material). Stabilized artificial saliva supplemented with mucin was obtained from Pickering Laboratories, Inc. (Mountain View, CA, United States). Normal saliva (Pooled human donors, pre-COVID) was obtained from Lee Biosolutions, Inc. (Maryland Heights, MO, United States).

TGX FastCast™ Acrylamide 10% kit, ammonium persulfate [(NH_4_)_2_S_2_O_8_], tetramethylethylenediamine (TEMED, C_6_H_16_N_2_), 2-mercaptoethanol, 10X Tris/Glycine/SDS buffer, 4X Laemmli sample buffer, Coomassie Brilliant Blue R-250 staining and destaining solution, and Precision Plus Protein™ Standard (10–250 kDa) were purchased from Bio-Rad Laboratories, Inc. (Hercules, CA, United States).

Octet^®^ Streptavidin (SA) biosensors were purchased from Sartorius AG (Germany), and black 96-well flat-bottom microplates (Greiner Bio-One) were purchased from Fisher Scientific (Waltham, MA, United States).

### Biomaterial Preparation

Electrophoresis in sodium dodecyl sulfate–polyacrylamide-based discontinuous gel (SDS-PAGE) was used to check the quality, purity, and size of recombinant proteins used in this study (Supplementary Figure S1). SDS-PAGE was performed using an acrylamide kit 10% (w/v). Ammonium persulfate 10% (w/v) and TEMED were added to catalyze gel polymerization. Protein solutions were denatured in 4X Laemmli sample buffer (ratio 9:1) supplemented with 2-mercaptoethanol 20% (v/v) and heated at 80°C for 10 min. The electrophoresis run was performed in Tris/Glycine/SDS 1X (v/v) running buffer at 200 V for 35 min, using the Mini-PROTEAN^®^ Tetra vertical electrophoresis cell system (Bio-Rad Laboratories, Inc., Hercules, CA, United States). After electrophoresis, the gel was stained in Coomassie Brilliant Blue R-250 staining solution for 40 min and destained in Coomassie Brilliant Blue R-250 destaining solution for 3 h to overnight. Precision Plus Protein™ standard (10–250 kD) was used to size the molecular weight of the proteins in GelAnalyzer 19.1 software (www.gelanalyzer.com, developed by Istvan Lazar Jr., PhD and Istvan Lazar Sr., PhD, CSc).

Truncated hACE2 was biotinylated with NHS-PEG4-Biotin reagent at a molar coupling ratio (MCR) of 1:1 according to biotin-reagent recommendations. The biotin reagent labels primary amino groups (−NH_2_) at the N-terminal and at the side chains of lysine (K) residues in the protein sequence. In brief, to modify hACE2 at 0.75 mg/ml stock concentration, 1.2 μl of 1 mM biotin-reagent solution was added to 130 μl of protein solution. The protein solution was incubated for 30 min at room temperature. The non-reactive biotin was removed using a desalting column.

Quantitative RT-PCR (qRT-PCR) was used to quantify the concentration of attenuated SARS-CoV-2 virus samples (Supplementary Material). A saliva diagnostic multiplex qRT-PCR, “TigerSaliva”, was performed at Clemson University Research and Education in Disease Diagnosis and Intervention (REDDI) Lab (CLIA # 42D2193465) (Ham et al., 2022). For this study, we modified the sample preparation steps slightly from the EUA-approved protocol [REF: Vogels]; the samples were diluted 10 times in RNAse-free water and then heated at 95°C for 10 minutes, instead of direct heating of the samples as per the EUA protocol. The TigerSaliva test measures the N1 sequence of SARS-CoV-2 (Primers: Integrated DNA Technology (IDT) 10006830 and 10006831; Probe: IDT 10006832) and Hs_RPP30 (human control gene) (Primers: IDT 10006836 and 10006837; Probe: IDT 10007062). The N-gene of SARS-CoV-2 is a single-copy gene, thus one copy of the N-gene is equivalent to one copy of the virus. Synthetic SARS-CoV-2 RNA was used for the calibration curve. For each replicate, 2 μl of diluted heat-treated samples was added to the reagents [New England Biolabs (NEB) Luna Buffer (M3006B), and Luna Enzyme Mix (M3002B)]. The amplification was conducted in a 384-well thermocycler (Bio-Rad CFX 384) plate according to the TigerSaliva multiplex protocol (Ham et al., 2022).

### Qualitative Screening of Binding by Biolayer Interferometry

Qualitative binding screening of biotinylated hACE2 toward UV-attenuated SARS-CoV-2 virions was performed with an OctetRED96 instrument (Sartorius, Inc.) at 30°C and 1,000 rpm sample agitation. Sodium (38.3 g/L) and sodium bicarbonate (11.6 g/L) physiological solution supplemented with 10% (v/v) stabilized artificial saliva and 0.01% (v/v) Tween 20™ (pH 7) was used as a binding buffer. Streptavidin (SA) biosensors were hydrated in a binding buffer for 600s. After hydration, tips were immersed in the binding buffer well to get the baseline for 60s. Next, tips were loaded with 10 μg/ml biotinylated hACE2 for 300s. In the association step, the loaded tips were exposed to the target molecules for 300s. After association with the target molecule, sensor tips were returned to the baseline binding buffer well for disassociation. Regression (hyperbolic) modeling for screening hACE2-SARS-CoV-2 interactions was calculated by fitting non-linear models to the experimental data using the approach by Krämer et al. (2019).

### Laser-Induced Graphene Electrode Fabrication and Selection of Replicates

LIG electrodes were fabricated on a Kapton film substrate using a CO_2_ laser (VLS2.30DT, Universal Laser Systems, Inc., Scottsdale, AZ, US) at 75% speed, 40% power, and 1000 PPI (Lin et al., 2014). The working electrode was designed in CorelDraw with a circular working area (***ϕ*** = 3.0 mm), connected to a stem (14.3 × 2.0 mm), that leads to a rectangular bonding pad (2.9 × 2.5 mm) (Supplementary Figure S2). A nitrocellulose passivation layer was applied on the stem area, and a metallic tape was incorporated on the bonding pad area for enhancing shear strength. The LIG electrodes were further modified with platinum nanoparticles (nPt) *via* electrodeposition by connecting the LIG electrode to the anode and a platinum wire to the cathode of a DC power supply (HM305P, HANMATEK). Next, LIG and Pt wire were immersed in a solution of 1.44% (v/v) chloroplatinic acid and 0.002% (v/v) lead acetate. The DC power supply was programmed to hold a constant potential of 10 V for 90s during the electroplating process.

Fabricated LIG-nPt electrodes were tested *via* electrochemical methods using a benchtop MultiPalmSens4 potentiostat (PalmSens, Houten, Netherlands) connected to a 3-electrode cell stand. The three-electrode system consisted of an Ag/AgCl (3M KCl) reference electrode (BASi^®^, West Lafayette, IN, United States), platinum wire auxiliary electrode (BASi^®^, West Lafayette, IN, United States), and LIG-nPt working electrode. Cyclic voltammetry was carried out in a solution containing KCl (100 mM), K_3_ [Fe(CN)_6_] (2.5 mM), and K_4_ [Fe(CN)_6_] (2.5 mM) using a potential range from −0.8 to 0.8 V at a scan rate of 200 mV/s for 10 cycles. The resulting voltammograms were used to select replicate LIG-nPt electrodes for further biofunctionalization and testing.

### Electrode Biofunctionalization

LIG-nPt electrodes were biofunctionalized with biotinylated hACE2 (LIG-nPt-hACE2) *via* streptavidin–biotin coupling. The appropriate electrode surface density of streptavidin (1.88 × 10^16^ units/mm^2^) and hACE2 (1.02 × 10^16^ units/mm^2^) was first determined *via* theoretical approximations and tuned through receptor loading experiments.

Since streptavidin has net negative surface charge, its immobilization on the electrode surface could be facilitated by electrostatic interactions. Prior to performing the biofunctionalization procedure, LIG-nPt electrodes were polarized *via* chronoamperometry at +1.0 V for 600s in a 2X (w/v) solution of sodium chloride (38.3 g/L)/sodium bicarbonate (11.6 g/L) buffer supplemented with 10% (v/v) pooled saliva (pH 8). Next, the electrodes were drop-casted with a suspension of his-tagged (6X-his) streptavidin (1 mg/ml), incubated for 10 minutes at room temperature, and rinsed with a buffer solution. While the polarization pretreatment was expected to expedite the deposition of streptavidin onto the LIG-nPt surface, stable surface coupling was achieved through histidine-platinum linking. Finally, the streptavidin-coated electrodes were drop-casted with a suspension of biotinylated hACE2 (0.75 mg/ml), incubated at room temperature for 10 minutes and rinsed with buffer solution.

### Electrode Microscopy Imaging

In order to demonstrate the platinum nanoparticles electrodeposition onto the LIG electrodes, a scanning electron microscopy analysis was performed using a scanning electron microscope SU5000 under the following conditions: 5 kV, 40 spot size, 50 Pa low vacuum, and magnification of 0.5, 2.5, 5, and 15 k.

To corroborate the presence and distribution of biotinylated hACE2 and recombinant S protein-RBD onto the LIG-nPt electrode surface, confocal microscopy images were taken on a Leica SPE confocal (Leica Microsystems, Wetzlar, Germany) at the Clemson Light Imaging Facility (Clemson Division of Research, Clemson University, Clemson SC, US). Biotinylated hACE2 was directly labeled with AlexaFluor™ 488 Microscale Protein Labeling Kit (Invitrogen™, green, λ 495/519 nm). Recombinant S protein-RBD was directly labeled with AlexaFluor™ 555 Microscale Protein Labeling Kit (Invitrogen™, orange/red, λ 555/565 nm).

### Electrochemical Studies

Electrochemical tests were carried out with a three-electrode cell stand (BASi^®^, C-3 Cell Stand, West Lafayette, IN, United States) connected to a benchtop potentiostat/impedance analyzer (PalmSens^®^, MultiPalmSens4, Houten, Netherlands). A platinum wire (7.5 cm) was used as auxiliary electrode (BASi^®^, MW1032, West Lafayette, IN, United States), Ag/AgCl (3M KCl) was used as reference electrode (BASi^®^, MF 2056, West Lafayette, IN, United States), and an ACE2-functionalized electrode (LIG-nPt-hACE2) was used as working electrode.

Electrochemical impedance spectroscopy (EIS) was performed in a non-Faradaic mode by immersing the electrodes in a 2X (w/v) solution of sodium chloride (38.3 g/L)/sodium bicarbonate (11.6 g/L) buffer supplemented with 10% (v/v) pooled saliva (pH 8) at 22°C and 1 atm. EIS spectra were obtained within the frequency range of 0.01 Hz–10,000 Hz, using an AC amplitude of 0.08 V and a DC voltage of 0.36 V.

Titration experiments were conducted to evaluate the EIS response of the LIG-nPt-hACE2 biosensors to clinically relevant concentration levels of SARS-CoV-2 in saliva (Bar-On et al., 2020). The analyte solution (attenuated virus suspended in pooled saliva) was drop-casted onto the working electrode and incubated for 10 min at room temperature. After incubation, the electrodes were rinsed and then tested using EIS.

### Biosensor Performance

Electrochemical impedance spectroscopy (EIS) tests yielded data on total impedance (Z), real impedance (Z′), imaginary impedance (Z″), series capacitance (Cs), real capacitance (C′), and imaginary capacitance (C″) at 63 cutoff frequencies within the 0.01 Hz to 10,000 Hz range (Sidhu et al., 2016; Oliveira et al., 2019; Sidhu et al., 2020; Giacobassi et al., 2021). Thus, EIS spectra were constructed based on the real and imaginary components of the complex capacitance according to Eqs 1, 2, respectively (Taberna et al., 2003; Randriamahazaka and Asaka, 2010; Yagati et al., 2020).

(1) $$C^{\prime }(\omega )=\frac{-Z^{\prime \prime }(\omega )}{\omega |Z(\omega ){|}^{2}},$$

(2) $$C^{\prime \prime }(\omega )=\frac{Z^{\prime }(\omega )}{\omega |Z(\omega ){|}^{2}},$$

where *C*′(*ω*) and *C*″(*ω*) are the real and imaginary parts of the complex capacitance spectra, *Z*′(*ω*) and −*Z*″(*ω*) are the real and imaginary parts of the complex impedance spectra, *Z*(*ω*) is the impedance modulus, and *ω* is the angular frequency.

Limit of detection (LOD) was calculated using the standard approach, with confidence varying from 99.7% (3 SD) to 94.5% (2 SD). Limit of blank (LOB) was calculated based on 90% confidence (1.645 SD). LOD, LOB, and limit of quantification (LOQ) were determined based on the approach described in Armbruster and Pry (2008). Non-specific binding in the electrochemical detection process was determined from matrix effects and non-targeted binding controls. Matrix effects were unveiled by recording the output of the biosensor exposed to a blank sample of pooled saliva. Non-target binding was explored by exposing the hACE2 biosensor to a negative control made of UV-attenuated hCoV-OC43 virus suspended in pooled saliva. The results from the matrix effects and negative control were contrasted with the response obtained from the biosensor’s exposure to a UV-attenuated variant of SARS-CoV-2.

## RESULTS AND DISCUSSION

### Binding Signatures of Truncated-Human Angiotensin-Converting Enzyme II With Attenuated Severe Acute Respiratory Syndrome Coronavirus 2 Virions

Analysis of binding affinity is important for understanding the molecular interactions between the ligand (hACE2) and target analyte (intact viral particle). Biolayer interferometry (BLI) is a label-free, real-time method for characterizing association and disassociation kinetics based on interferometric shift at the tip of a glass fiber sensor (Abdiche et al., 2008; Wilson et al., 2010). BLI does not require fluidics or control systems and is a relatively simple multiplexing system for screening ligand–analyte interactions. BLI produces chrono-interferometric plots for baseline drift, receptor loading, ligand–analyte association, and ligand–analyte disassociation. BLI time series plots were plotted for screening association rate (k_on_) and dissociation rate (k_off_). Under most conditions (>0.01 K_D_), k_off_ is not dependent on analyte concentration. Thus, the half-life is equal to the disassociation constant k_off_. Rather than performing detailed analysis of global binding affinity (K_D_) or apparent affinity (K_D_app_).

In our study, biolayer interferometry was used to screen binding signatures of truncated hACE2 with UV-attenuated SARS-CoV-2 wild type and its variants. We also benchmarked the test against two human coronaviruses (hCoV) responsible for common cold (namely hCoV-229E and hCoV-OC43).

**FIGURE 1 |:**
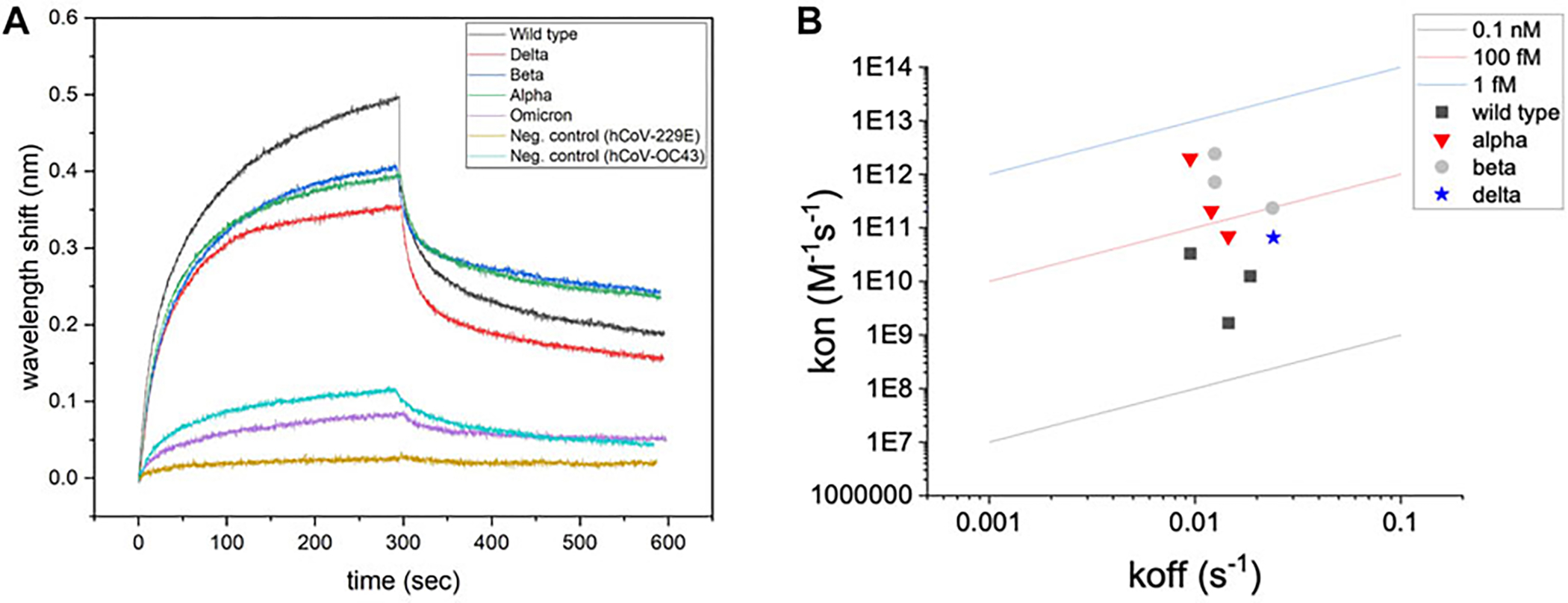
Screening of binding with biolayer interferometry. **(A)** Representative binding signatures of truncated hACE2 toward SARS-CoV-2 UV-attenuated virions (including wild type and four variants). hCoV-OC43 (common cold Betacoronavirus) and hCoV-229E (common cold Alphacoronavirus) were tested as negative controls for benchmarking. **(B)** Regression (hyperbolic) modeling for screening hACE2–SARS-CoV-2 interactions.

Regression (hyperbolic) modeling was used to characterize the association and disassociation curves for each test. Representative binding signatures of truncated hACE2 with UV-attenuated SARS-CoV-2 wild type and its variants, and the negative controls (Common cold viruses) are shown in Figure 1A. The binding toward wild type variant showed the highest interferometric shift (wavelength shift, nm), followed by Alpha and Beta variants, then Delta variant. The interaction with Omicron variant was similar to negative controls for both *Betacoronavirus* (hCoV-OC43) and *Alphacoronavirus* (hCoV-229E). An iso-affinity map (i.e., on–off interaction map) is shown in Figure 1B. Reference iso-affinity lines are shown for K_D_ values ranging from 0.1 nM to 1 fM. The interaction map indicates that the K_D_ values all lie within this range.

Binding toward a non-target *Betacoronavirus* is expected for hACE2 since SARS-CoV-2 and hCoV-OC43 belong to the same phylogenetic cluster (beta-CoV), sharing 28% of identity in the Spike protein sequence (Padhan et al., 2021). Instead, no association signals were detected for human coronavirus 229E strain, which belongs to a different phylogenetic cluster (alpha-CoV) than SARS-CoV-2. Computational modeling shows that some degree of similarity in the Spike protein structure is detected for human coronavirus belonging to the same phylogenetic cluster or lineage (Cueno and Imai, 2021).

### Biosensor Fabrication and Biofunctionalization

**FIGURE 2 |:**
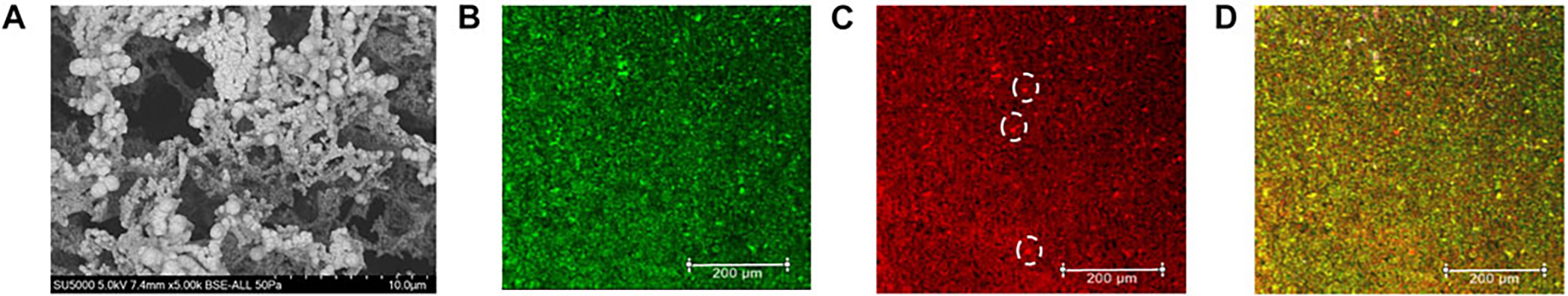
LIG-nPt electrode microscopy imaging. **(A)** Scanning electron microscopy (SEM) of LIG with platinum nanoparticles with a magnification of 5.0 k. **(B)** Confocal imaging of truncated hACE2 directly labeled with AlexaFluor™ 488 (Invitrogen™, green) showing the protein distribution on the LIG-nPt electrode surface. **(C)** Confocal imaging of S protein-RBD directly labeled with AlexaFluor™ 555 (Invitrogen™, orange/red) showing the target molecule distribution onto LIG-nPt electrode functionalized with hACE2. White dashed circles indicate microscale clumping that does not coincide with clumping observed in panel **(B)**. **(D)** Overlaid image of **(B,C)** showing the co-localization of the hACE2 and spike RBD in the working electrode. Imaging was performed using the Leica SPE confocal. Scale bars: 200 μm.

Figure 2A shows a representative SEM of a nanoplatinum-modified LIG (LIG-nPt) electrode. As demonstrated in our previous work (Chaturvedi et al., 2014; Vanegas et al., 2014; Taguchi et al., 2016), electrodeposition of nPt onto LIG increases the electroactive surface area (Supplementary Figure S3) and provides a more homogenous and net positive charge stable surface. The image shows an amorphous dendritic structure, which is similar to other images of LIG. Based on our previous work with nPt electrodeposited onto carbon electrodes, we assume the spherical structures are platinum, including fractal-like representations common to nPt-carbon.

The streptavidin (SAv)-biotin system was used for immobilizing hACE2 on the electrode. Electrode biofunctionalization with SAv-hACE2 and subsequent binding of S-protein were verified using confocal imaging (Figures 2B–D). SAv was immobilized *via* covalent binding between histidine (6X-his) and nPt according to our previous work (Rong et al., 2018). AlexaFluor 488–labeled 6x-his ACE2 (green channel) was then immobilized *via* streptavidin–biotin linking (Lue et al., 2004). Figure 2B shows a representative image of the LIG-nPt-hACE2 electrode surface (0.25 mm^2^ representative grid). Figure 2C shows a representative confocal image of AlexaFluor 555–labeled S protein RBD on the electrode surface (red channel). The dashed circles in panel **C** indicate microscale clumping of S protein that does not coincide with microscale localization of hACE2 observed in panel **B** (supplemental section for image analysis, Supplementary Figures S4, S5). This clumping of S protein may indicate a non-specific binding toward the LIG-nPt electrode surface. Figure 2D shows a composite image of the green and red channels (yellow indicates coincidence between hACE2 and S protein RBD). Overall, the images indicate that the his-SA + biotinylated hACE2 immobilization was successful, and hACE2-coated electrodes had affinity toward S protein RBD under these conditions. Heterogenous clumping (bright green and red spheres in panel **D**) was observed in approximately 3% of the samples, which may be due to areas of high nPt deposition [supplemental for image analysis using methods in our previous work (Hills et al., 2018; Giacobassi et al., 2021)].

**FIGURE 3 |:**
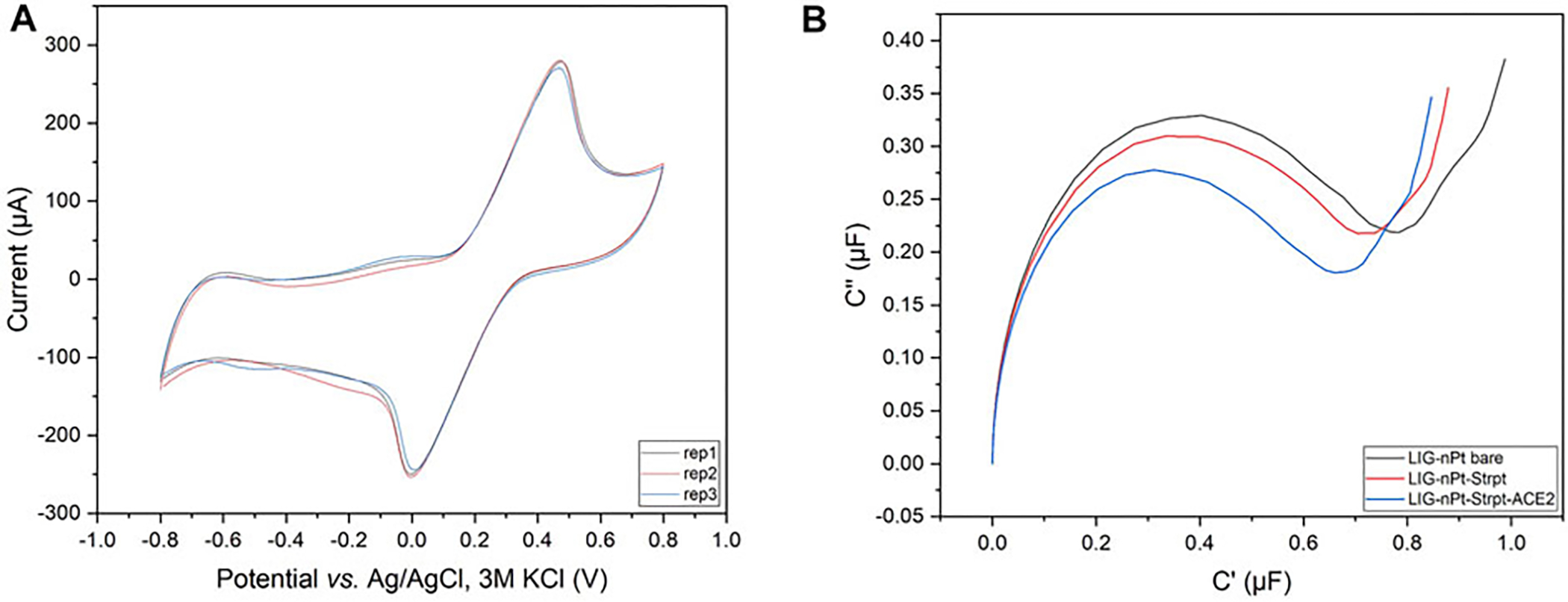
Replicate selection and biofunctionalization. **(A)** Representative cyclic voltammogram of the LIG-nPt electrode replicates vs. Ag/AgCl (3M KCl) in 100 mM KCl and 2.5 mM K_3_ [Fe(CN)_6_]/K_4_ [Fe(CN)_6_] at scan rates of 200 mV s^−1^. **(B)** Representative Nyquist capacitive plot (C′ vs. C″) of biofunctionalization with truncated hACE2 biotin-tag immobilized on the working electrode *via* the streptavidin–biotin coupling method.

Cyclic voltammetry (Faradaic) at 200 mV/s was used as quality control (QC) criteria for selecting replicate LIG-nPt electrodes. Figure 3A shows a representative example of triplicate LIG-nPt electrodes selected from the large fabrication batch (*n* = 35). The QC screening criteria reduced the variation to 1.5% (supplemental section for details, Supplementary Figure S6). After selecting triplicate LIG-nPt electrodes, triplicate sets were biofunctionalized with streptavidin and hACE2.

EIS was used to analyze baseline LIG-nPt electrodes before and after biofunctionalization. LIG biosensors have been developed based on either impedance (Soares et al., 2020; Wang G. et al., 2021) or capacitance (Arya et al., 2018; Yagati et al., 2020). To determine the most appropriate response variable under the conditions tested here, impedance (net, real, imaginary) and capacitance (series, real, imaginary) were both analyzed. Figure 3B shows data from LIG-nPt electrodes, demonstrating a well-characterized capacitive signal response during biofunctionalization. Similar to recent work in capacitive biosensing with LIG (Yagati et al., 2020), the representative Nyquist capacitive plot (C′ vs. C″) in the Figure 3B shows a clear signal change during biofunctionalization with streptavidin and hACE2 layers on LIG-nPt. Thus, imaginary capacitance (C″) was used as the response variable for development of the bioassay.

### Biosensor Performance

**FIGURE 4 |:**
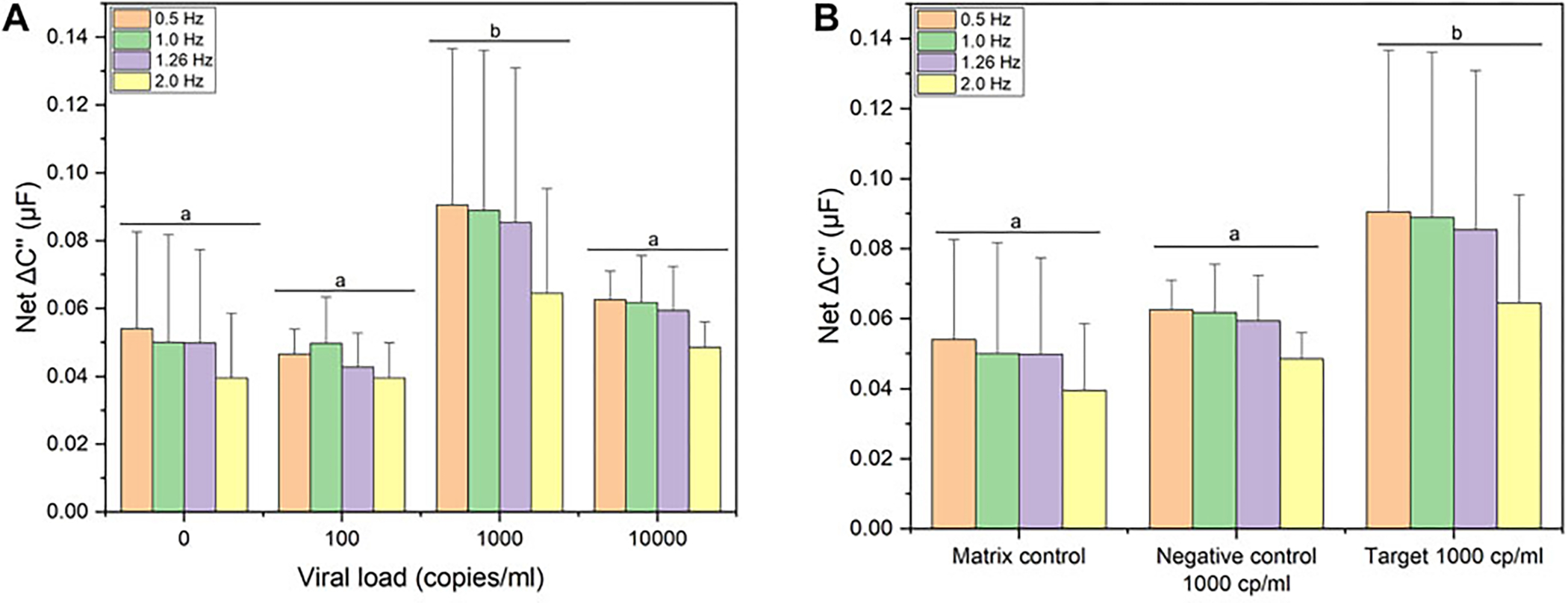
Electrochemical detection studies. **(A)** Signal response following incubation with 0, 100, 1000, and 10000 copies/mL of UV-attenuated SARS-CoV-2 Delta variant. **(B)** Signal response following incubation with clinical relevant viral load (1000 copies/ml) of UV-attenuated SARS-CoV-2 Delta variant; negative control: UV-attenuated human coronavirus (OC43) and matrix effects (pooled saliva target-free). Four optimum cutoff frequencies are shown for each data set. Error bars represent standard deviation from the mean measurement (*n* = 12 for matrix control; *n* = 3 for negative control and target). Same letters represent groups with no significant difference (*p* < 0.05).

The analytical sensitivity of the biosensor toward UV-attenuated SARS-CoV-2 Delta variant is shown in the Figure 4A. Capacitance spectra from 0.01 Hz to 10 kHz (Supplementary Figure S7) were analyzed for feature reduction. A select number of optimum cutoff frequencies for each data set is shown. Titration shows a clear response (net ΔC″) for a viral load of 1,000 copies/ml. However, at high titer (10,000 copies/ml) hACE2 is likely displaced due to competition, indicated by the decrease in signal at all frequencies shown in Figure 4A. In other words, affinity between hACE2 and DELTA is on the order of SAv-biotin for immobilizing hACE2. This is supported by the apparent K_D_ values shown in the iso-affinity map (K_D_ in the 0.1 nM to 1 fM range) compared to the affinity of SAv-biotin affinity (approximately 100 to 1,000 fM).

Based on the hACE2 biosensor response to a viral load of 1,000 copies/ml, the biosensor was benchmarked against the same viral load of a non-target human coronavirus (hCoV-OC43) as well as a matrix control (target-free pooled saliva). Figure 4B shows the capacitive signal (net ΔC″) toward lower threshold viral load (1,000 copies/ml) of SARS-CoV-2 Delta variant (noted as “target”) is shown in comparison to hCoV-OC43 (shown as “negative control”) along with pooled saliva (noted as “matrix control”. Based on a multivariate analysis (lumped optimum cutoff frequencies), the capacitance change for SARS-CoV-2 Delta variant was significantly different (ANOVA/Tukey, *p* < 0.05) to the matrix control and negative control. This affinity is confirmed by the BLI binding signatures (Figure 1A), as both tests show cross-binding of hACE2 toward hCoV-OC43, a *Betacoronavirus*. However, we were able to differentiate the capacitance response between target and non-target coronavirus showing the specificity of the hACE2 biosensor toward SARS-CoV-2.

The total test time was under 30 min (10 min for data collection/analysis, estimated sample collection and handling time of 10 min). Calibration plots toward SARS-CoV-2 (DELTA variant) in pooled saliva were prepared to calculate key performance indicators (supplemental section for details, Supplementary Figure S8), using the optimal cutoff frequency of 0.5 Hz. The response was linear up to 1,000 copies/ml (R^2^ = 0.98). The limit of blank (LOB, 1.645 SD) was 65 copies/ml. At 99.7% confidence (3SD), the limit of detection (LOD) was 2,960 copies/ml. This value is larger than the linear operating range, indicating that LOD must be reported at a lower confidence interval. When the confidence is reduced to 95.4% (2SD), the LOD was 990 copies/ml, within the linear operating range. Applying this calibration curve to target and matrix control samples, the biosensor was precise up to 1,000 but lacked accuracy when exposed to other coronaviruses (false positives). Based on this result, the technology was analyzed as a quantitative detector using standard approaches in the literature.

**FIGURE 5 |:**
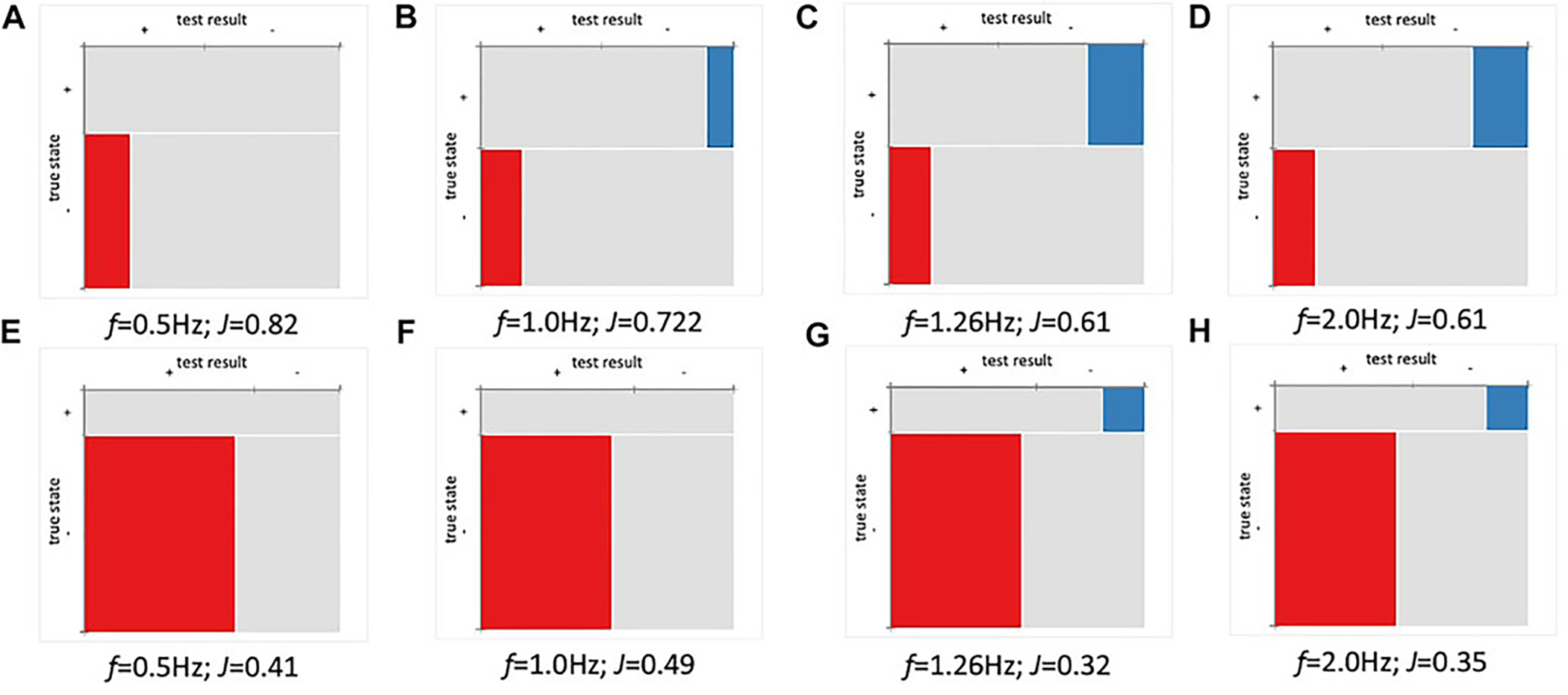
Positive percent agreement (PPA) and negative percent agreement (NPA) of ACE2 detector for SARS-CoV-2 (Delta) at various frequencies (*n* = 36). Youden index (J) and cutoff frequency (*f*) shown for each plot. **(A–D)** Mosaic plot for detection of Delta variant (0–1,000 copies/ml). **(E–H)** Mosaic plot for Delta (0–1,000 copies/ml) variant including matrix and negative controls. The threshold was set using baseline (μ±1SD).

Using a threshold of 1,000 copies/ml set by the Centers for Disease Control (CDC) as a threshold for SARS-CoV-2 detection (Arnaout et al., 2020), data were converted to binary output for calculation of analytical sensitivity, positive percent agreement (PPA) and negative percent agreement (NPA). Based on the approach in Figure 4, the analysis analyzed imaginary capacitance (C″) at various cutoff frequencies (*f*). Figures 5A–D shows Mosaic plots for detection of DELTA variant using hACE2 biosensors in 10% pooled saliva over the concentration range of 0–1,000 copies/ml (UV-attenuated DELTA variant). The highest Youden index (J) was at 0.5 Hz, with decreasing index score as *f* increased to 2.0 Hz. Analysis of high titer (10,000 copies/ml) is not shown here due to displacement of hACE2 as previously noted. In Figure 5A, two blank samples showed a false positive (red box in Mosaic plot), but these were within 5% of the threshold and could be excluded with a more advanced data classification approach. Analysis at higher cutoff frequency showed both false positive and false negative in the absence of matrix controls and negative controls (Figures 5B–D). When including matrix and negative controls in the binary analysis (Figures 5E–H), the false-positive ratio increases significantly (likewise the j value decreases to 0.42). This is expected, and in fact was conceived in the design, as hACE2 was used as a generalist biosensor that screens variants as well as other similar coronaviruses. The highest J for this panel was at f = 1.0 Hz, however, this cutoff frequency may not be optimal since the detection of DELTA variant without controls (Figure 5B) showed false negatives. The optimal cutoff frequency of 0.5 Hz was used for this analysis.

Current SARS-CoV-2 biosensors based on hACE2 are shown in Table 1. Among these, EIS is the most common approach for signal transduction. EIS is sensitive to small changes in the dielectric layer near the sensor surface (Kumar et al., 2022), but care must be taken when selecting cutoff frequency for univariate analysis (Lindholm-Sethson et al., 2007). The technologies in Table 1 utilize truncated hACE2, which could be a limitation for future reproducibility at large scale. To date, the reported hACE2 biosensor describes a limit of detection in the range of femtogram to nanogram when targeting recombinant spike protein. A lower limit of detection of 38.6 copies/ml has been described for attenuated virus (wild type) (Vezza et al., 2021). In addition, it is important to notice that the mentioned study used a commercially printed circuit board (PCB) electrode modified with gold. Here, our limit of detection for attenuated virus ranges from 990 copies/ml (95.4% confidence, 2SD) to 2,960 copies/ml (99.7% confidence, 3SD), and 65 copies/ml (1SD) for limit of blank.

Our work is one of the few to fabricate the electrode itself with an easy-to-produce method based on an affordable material that is widely available (commercial polyimide). Amongst the advantages of using LIG over commercial electrodes, we can cite the following: 1) Customizable design of the electrodes’ size and shape: the electrode can be digitally designed according to the project needs using any vector graphics software (e.g., CorelDRAW). The CO_2_ laser can inscribe any pattern of the polymeric surface with micrometer resolution (Ye et al., 2019; Soares et al., 2020); 2) Tunable electrode properties: by adjusting the laser inscribing parameters it is possible to modify material properties such as electrical conductivity, surface morphology, and surface wettability without the need for post-chemical modification (Ye et al., 2019; Chen et al., 2022; Hjort et al., 2022); 3) Simplified manufacturing: LIG electrodes fabrication is a one-step process based on CO_2_-laser inscribing on a polyimide substrate. Fabricated electrodes can be further functionalized with nanoparticles and biomaterials according to the project needs (Ye et al., 2019); 4) Low-cost: the lab manufacturing cost of the LIG electrodes described in this work is less than $1 USD a piece. On the other hand, commercial carbon or platinum disk electrodes have an average cost of $200 USD per electrode; 5) Ecologically friendliness: while screen printed electrodes can be purchased at relatively low-cost, the screen-printing process uses toxic reagents and generates significant toxic waste from excess inks removal. On the other hand, the laser inscribing process does not require external reagents, and the LIG material exhibits low toxicity in aquatic organisms (d’Amora et al., 2020); 6) Disposability: considering the application of the biosensor developed in this work (detection of a highly transmissible coronavirus), it is important to safely dispose of any materials that get in contact with a potentially positive sample. The low-cost and flexible material of the LIG biosensors allow for post-use compact collection, sterilization, and disposal as solid waste. In addition, Pt nanoparticles electroplating onto LIG is an efficient and easily reproducible procedure (McLamore et al., 2011; Vanegas et al., 2014; Taguchi et al., 2016). Altogether, the ease-of-production, label-free format, simplicity to modify the biorecognition element, and the response to a threshold for detection indicate that the hACE2 biosensor shows promise as a point-of-need detection for future outbreaks.

In addition, the technologies describe in Table 1 were tested in nasopharyngeal/oropharyngeal and saliva samples. Our hACE biosensor was designed for saliva testing. Saliva is a complex and variable bio-mixture with potential diagnostic value for coronaviruses (Xu et al., 2020). SARS-CoV-2 has been detected by RT-PCR in saliva specimens (including for asymptomatic cases) and has showed less variability in the virus levels than nasopharyngeal swab specimens (Wyllie et al., 2020). Since saliva is a complex bio-mixture, we performed control experiments to check matrix effects on the biosensor surface. A non-specific binding between the complex components (such as mucin) and the hACE2 biosensor surface causes a capacitance change in response to interference from matrix. The reported biosensor for saliva detection (Table 1) do not make clear the existence of matrix effects from the saliva (target-free) on the biosensor surface. Beyond the matrix effects, our findings show that the detection of the target was significantly different from the matrix control. This suggests that non-specific binding from the matrix does not compromise analytical sensitivity. Our results provide support for potential use of saliva as a biological fluid for electrochemical sensing.

## CONCLUSION

We developed an impedimetric hACE2 biosensor for SARS-CoV-2 detection in saliva. The detection principle is based on the spike protein and human ACE2 interactions. Since hACE2 is the first point of interaction for SARS-CoV-2 infection, the hACE2 biosensor is responsive to mutations on the spike protein, being able to detect its variants. The biosensor is based on an accessible material, a simple one-step fabrication method, and a label-free format. The performance of the biosensor was by the capacitance response. The biosensor was able to detect SARS-CoV-2 into the lower defined threshold for qRT-PCR showing promise for detection of asymptomatic cases or lower viral load cases as well. The biosensor shows analytical sensitivity to a lower viral load (LOD ranges from 990 to 2,960 copies/ml), and specificity to SARS-CoV-2 when benchmarked with a human *Betacoronavirus* (common cold virus). Despite the background signal from saliva in the electrochemical sensing, the hACE2 biosensor was able to differentiate the capacitance change in response to target (Delta variant). Our findings show that saliva may be suitable as a non-invasive biofluid for electrochemical sensing.

## Supplementary Material

Supplementary Materials

## Figures and Tables

**TABLE 1 |:** Summary of ACE2-based biosensor for SARS-CoV-2 detection.

Approach	Biorecognition scheme	Sample(s) tested	Analytical target	LOD	Reference

Electrochemical impedance spectroscopy (EIS)	hACE2 immobilized on a commercial gold screen-printed electrode modified with EDC/NHS	Nasopharyngeal/ oropharyngeal samples	Recombinant spike protein (wild type)	299.30 ng ml^−1^	Büyüksünetçi et al. (2022)
Square wave voltammetry (SWV)	hACE2 immobilized on a graphite pencil electrode modified with gold nanoparticles	Clinical saliva and nasopharyngeal/oropharyngeal samples	Recombinant spike protein (wild type)	229 fg ml^−1^	Lima et al. (2021)
Electrochemical impedance spectroscopy (EIS)	hACE2 immobilized on a gold electrode modified with perfluorodecanethiol (PFDT) self-assembled monolayers (SAMs)	Saliva samples	Recombinant spike protein and inactivated virus (wild type/inactivated SARS-CoV-2 molecular standard kit)	1.68 ng ml^−1^ and 38.6 copies/ml	Vezza et al. (2021)
Electrochemical impedance spectroscopy (EIS)	hACE2 immobilized by streptavidin-biotin affinity on a laser-induced graphene (LIG) electrode modified with platinum nanoparticles	Pooled human saliva	S protein on UV-attenuated virus (Delta variant)	2,960 copies/ml* at 99.7% confidence (3SD)	This study

* The concentration of attenuated virion is based on RT-PCR analysis targeting ORF1a.

## Data Availability

The original contributions presented in the study are included in the article/Supplementary Material; further inquiries can be directed to the corresponding author.
