# Supplementary material for: Development of a Biosensor Based on Angiotensin-Converting Enzyme II for Severe Acute Respiratory Syndrome Coronavirus 2 Detection in Human Saliva
Source: Front Sens (Lausanne). Author manuscript; Available in PMC 2022 Aug 18. (PMC9386735; doi:10.3389/fsens.2022.917380)
Supplement: Supplementary Materials [file NIHMS1827456-supplement-Supplementary_Materials.docx]

Supplementary Material


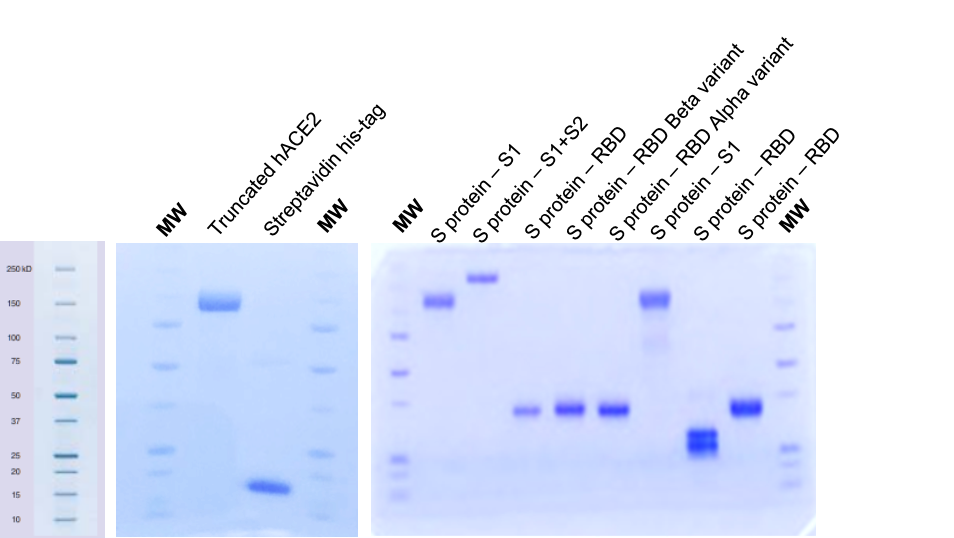


**Supplementary Figure 1.** SDS-PAGE analysis for quality control of commercial recombinant proteins. Protein electrophoresis analysis was performed with 10% Polyacrylamide at 200V for 30 minutes, stained with Coomassie R-250 for 40 minutes and destained overnight. A protein standard (10 to 250 kD) was used to size the protein molecular weight. MW: molecular weight pattern.

**
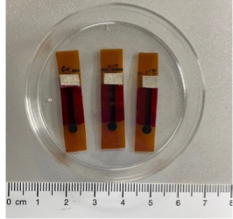
**

**Supplementary Figure 2.** Representative imagens of LIG-nPt electrodes fabricated on a Kapton film substrate using a CO_2_ laser at 75% speed, 40% power, and 1000 PPI. The working has a circular working area (𝝓=3.0 mm), connected to a stem (14.3x2.0 mm), and a rectangular bonding pad (2.9x2.5 mm).


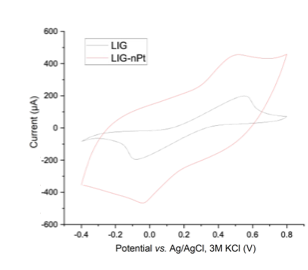


**Supplementary Figure 3.** Representative cyclic voltammogram of the LIG electrode before (LIG) and after (LIG-nPt) Platinum nanoparticles (nPt) electrodeposition vs. Ag/AgCl (3M KCl) in 100mM KCl, 2.5mM K_3_[Fe(CN)_6_]/K_4_[Fe(CN)_6_] at scan rates of 200 mV s^-1^.

**
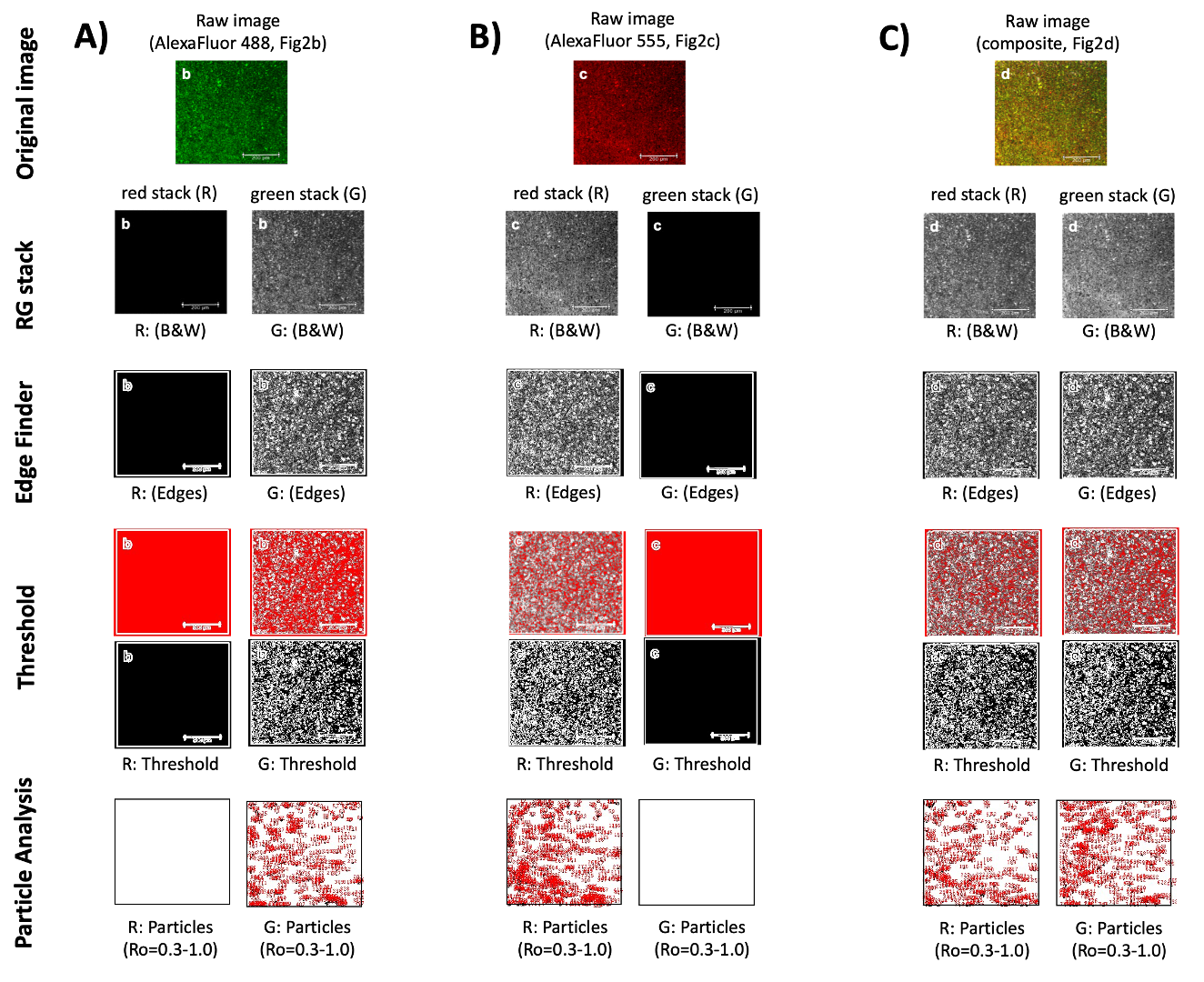
**

**Supplementary Figure 4.** Analysis of confocal images using ImageJ. RGB images were pixelated and R-G stacked (220x202 pixels; 8-bit). Edge location was then applied, followed by binary using a circularity coefficient (Ro) ranging from 0.3 (min) to 1.0 (max). Particle analysis is shown in the bottom row of images for all images. A) AlexaFluor 488 labeled 6x-his tagged hACE2 on LIG-nPt electrodes. B) AlexaFluor 555 labeled S protein RBD interacting with hACE-modified electrodes. C) Composite image derived from panels A and B.


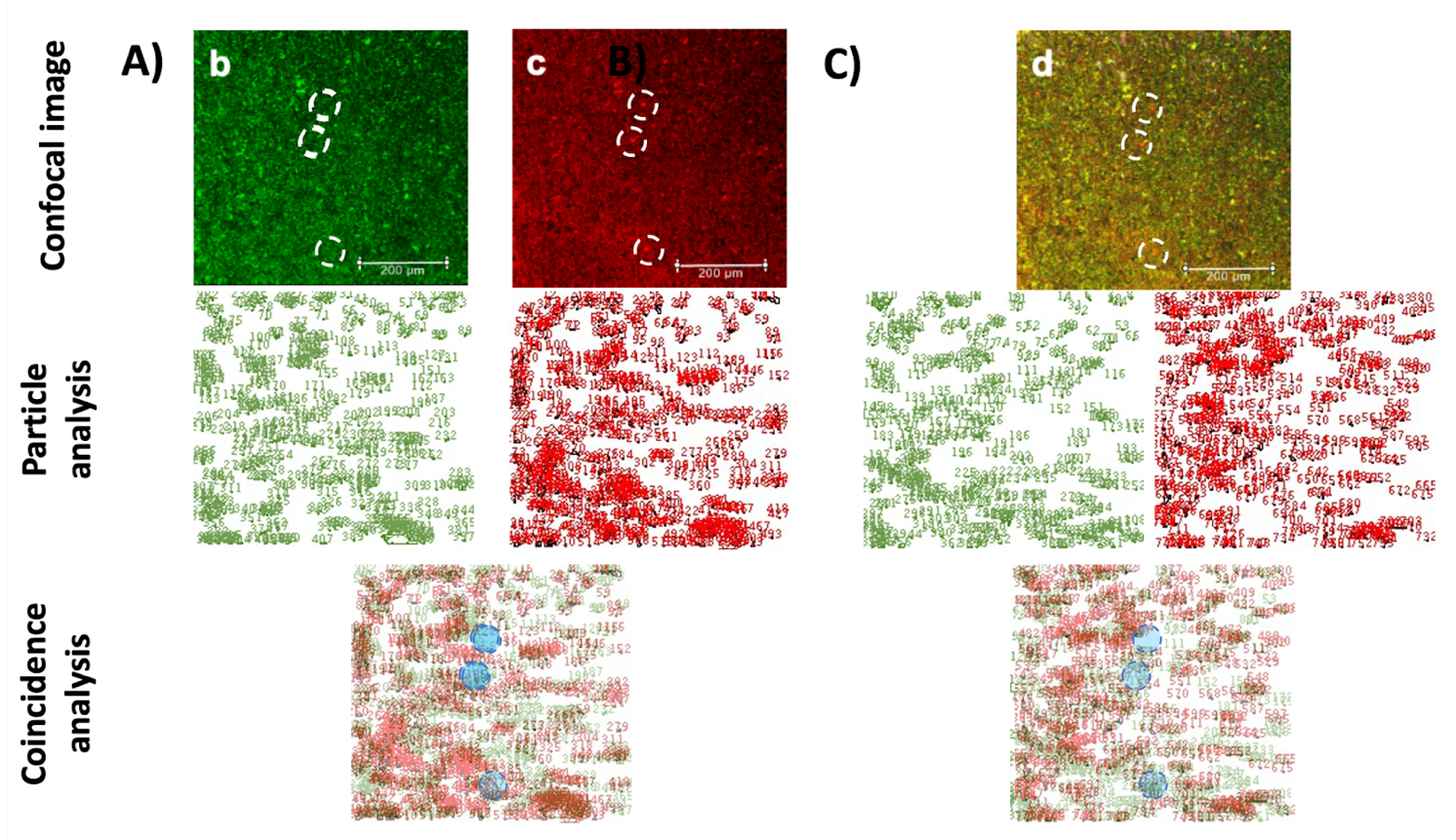


**Supplementary Figure 5.** Coincidence analysis based on ImageJ particle analysis. Top row: Confocal images with dashed circles indicated areas with no coincidence between green and red particles. Middle Row: Green and red stack images (binary) were recolored for coincidence analysis. Bottom row: Coincidence analysis in indicated by blue circles. Coincidence testing was used to identify red pixels that were not associated with green pixels (i.e., AlexaFluor 555 tagged S protein RBD that was not associated with his-tagged hACE2).


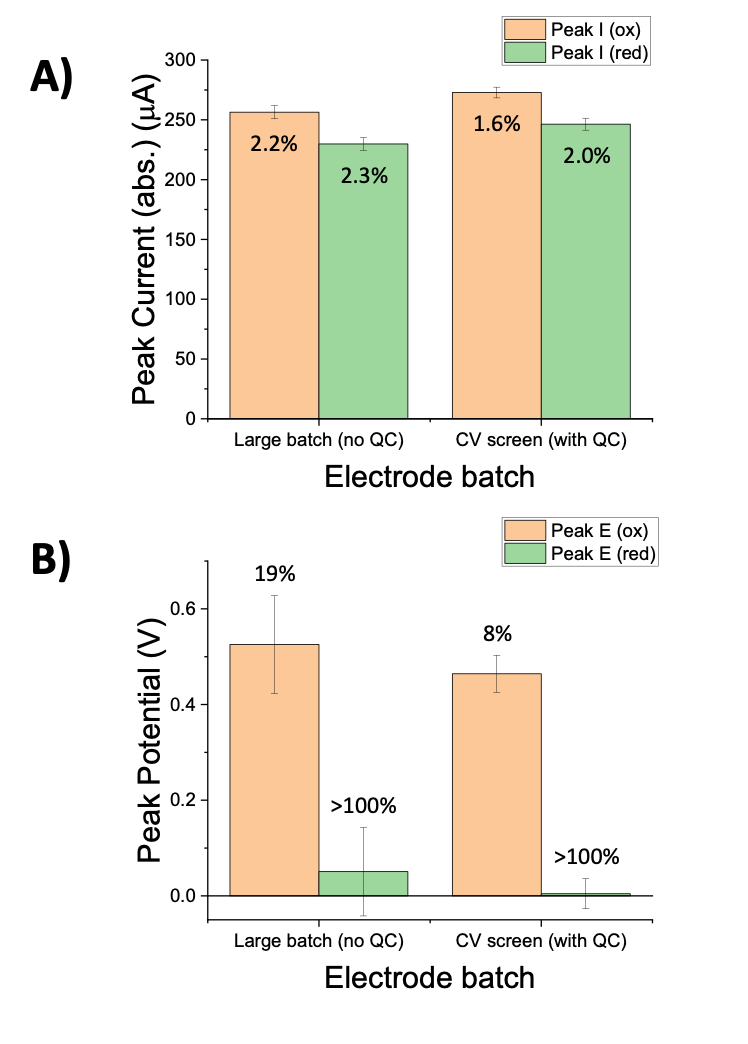


**Supplementary Figure 6.** Electrode selection from large batch fabrication for A) peak current and B) peak potential. The left vertical bars in each plot indicate large batches of fabricated LIG electrodes (n=35), right bars represent a pool of LIG electrodes after quality control screening (n=11). Error bars represent standard error of the arithmetic mean; coefficient of variation (%) is shown for each group.

**Supplementary Figure 7.** Representative Nyquist capacitive plot (C’ vs. C”) of complex capacitance spectra from 0.01Hz to 10kHz for UV-attenuated SARS-CoV-2 Delta variant viral load of (A) 0 copies/mL; (B) 100 copies/mL; (C) 1000 copies/mL; and (D) 10,000 copies/mL.


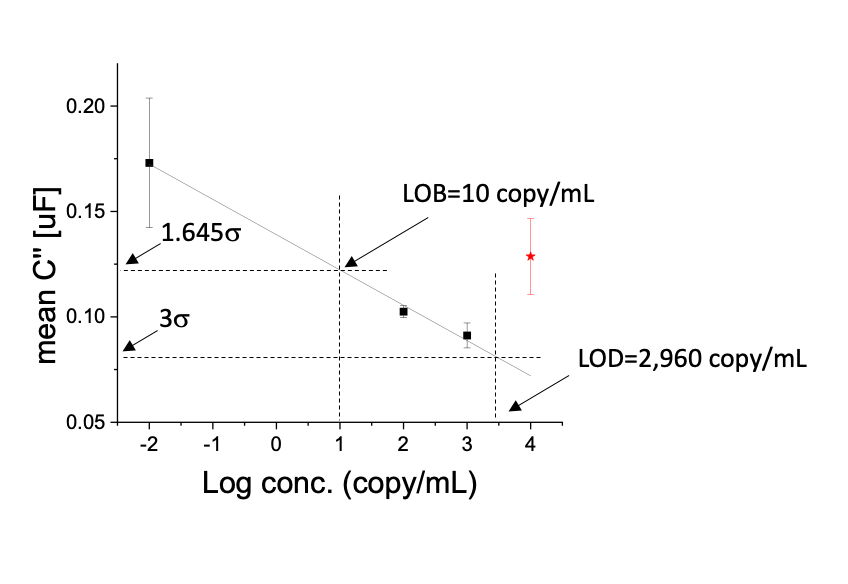


**Supplementary Figure 8.** Calibration of hACE2 LIG sensor toward SARS-CoV-2 (DELTA variant) in saliva. Limit of blank (LOB) and limit of detection (LOD) are indicated on the plot, along with the corresponding virion concentration. The red datapoint (star) is beyond the linear working range (concentration of 10,000 ORF1a/mL). The optimal cutoff frequency of 0.5 Hz was used for the calibration curve.

**Supplementary Table 1.** Summary of UV-attenuated virus samples used in this study. The virus samples were obtained from the University of California San Diego through the NIH RADx-rad Diagnostics Core Center (DCC).

| **Virus type** | **Virus isolate** | **Source** | **Concentration (copies/ml)** |
| --- | --- | --- | --- |
| SARS-CoV-2 wild type | SARS-CoV-2, Isolate USA-WA1/2020 - Washington strain | BEI Resources | 1.92x10^8^ (ORF1a/ml) |
| SARS-CoV-2 Beta variant | SARS-CoV-2, Isolate hCoV-19/South Africa/KRISP-K005325/2020 | BEI Resources | 5.60x10^7^ (ORF1a/ml) |
| SARS-CoV-2 Alpha variant | SARS-CoV-2, Isolate hCoV-19/USA/CA_UCSD_5574/2020 | UCSD Laurent’s and Carlin’s BSL3 Lab | 1.59x10^8^ (ORF1a/ml) |
| SARS-CoV-2 Delta variant | SARS-CoV-2, Isolate hCoV-19/USA/PHC658/2021 (Delta variant) | BEI Resources | 1.98x10^8^ (ORF1a/ml) |
| SARS-CoV-2 Omicron variant | SARS-CoV-2, Isolate hCoV-19/USA/CA-SEARCH-59467/2021 (Omicron variant) | UCSD Laurent’s and Carlin’s BSL3 Lab | 6.60x10^7^ (ORF1a/ml) |
| hCoV-229E | Human Coronavirus strain 229E | ATCC | 4.37x10^8^  (M gene/ml) |
| hCoV-OC43 | Human Coronavirus strain OC43 | ATCC | 2.49x10^5^ (Glycoprotein RNA/ml) |

**Supplementary Table 2.** Quantification of SARS-CoV-2 samples by qRT-PCR targeting N1 gene. Cycle threshold (Ct) values are showing for duplicates per plate (n=6).

| **Sample** | **C_q_ values for N1** | | | | | | | | **Mean N1 C_q_** |
| --- | --- | --- | --- | --- | --- | --- | --- | --- | --- |
|  | Plate 1 | | Plate 2 | | | Plate 3 | | |  |
| Wild type | 12.81 | 10.85 | | 13.67 | 19.03 | | 12.69 | 12.92 | 13.66 |
| Delta | 14.07 | 14.02 | | 15.55 | 15.40 | | 14.68 | 14.70 | 14.73 |
| Gamma | 15.03 | 14.90 | | 16.39 | 16 | | 15.24 | 15.70 | 15.54 |
| Control | 24.17 | 23.81 | | 25.49 | 26.08 | | 24.95 | 24.87 | 24.89 |

**Supplementary Table 3.** Prior to electrode quality control screening using Faradaic cyclic voltammetry. Error was approximated via standard error to account for variations in sample size. Coefficient of variation is shown as a percent of arithmetic mean.

| **Parameter** | **Large batch**  **(n=35)** | **Quality Control batch**  **(n=11)** |  |
| --- | --- | --- | --- |
| Peak Oxidation Current  [uA] | 256 +/- 6  (Coef. Var. 2.2%) | 273 +/- 4  (Coef. Var. 1.6%) | |
| Peak Reduction Current  [uA] | 230 +/- 6  (Coef. Var. 2.3%) | 246 +/- 5  (Coef. Var. 2.0%) |  |

**Supplementary Table 4.** Aggregated data for SARS-CoV-2 (DELTA) detection in saliva (concentration data are shown as (µ ± 1SD). False positive in matrix control (pooled saliva) and Betacoronavirus hCoV-OC43 (1000 copies/mL).

| **Analyte** | **Test Concentration (predicted)**  **[copies/mL]** | **True Concentration**  **[copies/mL]** |
| --- | --- | --- |
| Baseline | 1.5 ± 2.4 | 0 |
| Matrix control | 329 ± 326 | 0 |
| hCoV-OC43  (1000 cp/ml) | 2091 ± 2765 | 0 |
| SARS-CoV-2 Delta | 159 ± 66 | 100 |
| SARS-CoV-2 Delta | 1506 ± 1351 | 1,000 |
| SARS-CoV-2 Delta | 18 ± 19 | 10,000 |
